# 1-Hexadecyl-3-methyl­imidazolium bromide monohydrate

**DOI:** 10.1107/S1600536809014950

**Published:** 2009-04-30

**Authors:** Zengbin Wei, Xilian Wei, Shizhou Fu, Jie Liu, Daoxi Zhang

**Affiliations:** a College of Chemistry and Chemical Engineering, Liaocheng University, Shandong 252059, People’s Republic of China

## Abstract

In the crystal structure of the title compound, C_20_H_39_N_2_^+^·Br^−^·H_2_O, the 1-hexa­decyl-3-methyl­imidazolium cations are stacked along the *b* axis, forming channels parallel to [100] which are occupied by the bromide anions and water mol­ecules. The crystal is stabilized by O—H⋯Br, C—H⋯O and C—H⋯Br hydrogen-bonding inter­actions, generating a two-dimensional network.

## Related literature

For the applications of imidazolium compounds, see: Downard *et al.* (2004 ▶); Wasserscheid & Keim (2000 ▶). For the structure of free imidazole, see: Craven *et al.* (1977 ▶).
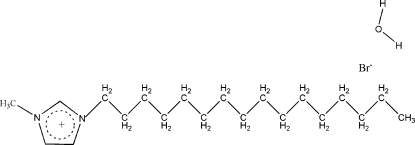


## Experimental

### 

#### Crystal data

C_20_H_39_N_2_^+^·Br^−^·H_2_O
*M* *_r_* = 405.46
Triclinic, 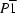

*a* = 5.4989 (5) Å
*b* = 7.8507 (9) Å
*c* = 27.330 (3) Å
α = 94.080 (1)°
β = 91.492 (1)°
γ = 101.929 (2)°
*V* = 1150.4 (2) Å^3^
*Z* = 2
Mo *K*α radiation
μ = 1.80 mm^−1^
*T* = 293 K
0.38 × 0.23 × 0.12 mm

#### Data collection

Bruker SMART CCD area-detector diffractometer
Absorption correction: multi-scan (*SADABS*; Sheldrick, 1996 ▶) *T*_min_ = 0.549, *T*_max_ = 0.813
5929 measured reflections
3995 independent reflections
3058 reflections with *I* > 2σ(*I*)
*R*_int_ = 0.026

#### Refinement

*R*[*F* ^2^ > 2σ(*F* ^2^)] = 0.057
*wR*(*F* ^2^) = 0.148
*S* = 1.04
3995 reflections
219 parameters
H-atom parameters constrained
Δρ_max_ = 0.58 e Å^−3^
Δρ_min_ = −0.31 e Å^−3^

### 

Data collection: *SMART* (Siemens, 1996 ▶); cell refinement: *SAINT* (Siemens, 1996 ▶); data reduction: *SAINT*; program(s) used to solve structure: *SHELXS97* (Sheldrick, 2008 ▶); program(s) used to refine structure: *SHELXL97* (Sheldrick, 2008 ▶); molecular graphics: *SHELXTL* (Sheldrick, 2008 ▶); software used to prepare material for publication: *SHELXTL*.

## Supplementary Material

Crystal structure: contains datablocks I, global. DOI: 10.1107/S1600536809014950/rz2309sup1.cif

Structure factors: contains datablocks I. DOI: 10.1107/S1600536809014950/rz2309Isup2.hkl

Additional supplementary materials:  crystallographic information; 3D view; checkCIF report

## Figures and Tables

**Table 1:** Hydrogen-bond geometry (Å, °)

*D*—H⋯*A*	*D*—H	H⋯*A*	*D*⋯*A*	*D*—H⋯*A*
O1—H1*C*⋯Br1^i^	0.85	2.58	3.429 (4)	180
O1—H1*D*⋯Br1^ii^	0.85	2.52	3.373 (4)	180
C3—H3⋯Br1^iii^	0.93	2.75	3.661 (4)	167
C1—H1⋯O1^iv^	0.93	2.38	3.231 (6)	153

Symmetry codes: (i) 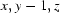; (ii) 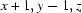; (iii) 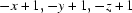; (iv) 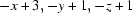.

**Fig. 1.**
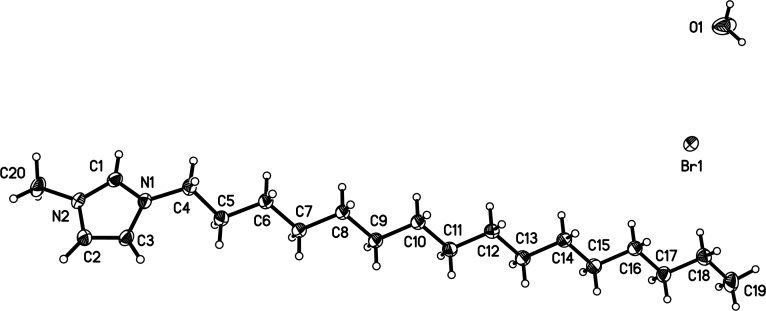
The molecular structure of the title compound, with atom labelling scheme and 50% probability displacement ellipsoids.

**Fig. 2.**
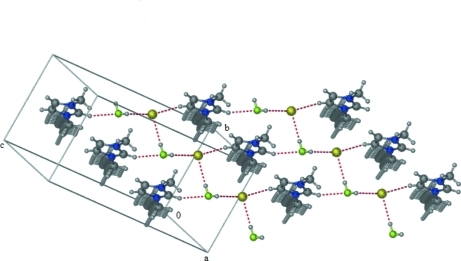
Crystal packing of the title compound, showing the two-dimensional network structure formed by O—H···Br, C—H···O and C—H···Br hydrogen bonds (dashed lines).

## supplementary crystallographic information

### Comment

Various ionic liquids based on imidazolium cations such as 1-alkyl-3-methylimidazolium have been extensively investigated over the last several years (Wasserscheid & Keim, 2000), in particular with respect to their applications as liquid crystals (Downard *et al.*, 2004). As a contribution to the chemistry of ionic liquids, we report here the synthesis and crystal structure of the title compound.

The asymmetric unit of the title compound (Fig. 1), consists of a 1-hexadecyl-3-methylimidazolium cation, a bromide anion and a water molecule. The C1—N1—C3 bond angle of 108.0 (3)° is similar to those in free imidazole (Craven *et al.*, 1977). The relative orientation of the imidazolium ring with respect to the aliphatic chain can be described by the value of -126.4 (4)° of the C1—N1—C4—C5 torsion angle. The N1—C4 bond length is 1.472 (5) Å. In the crystal, the cations are stacked along the *b* axis forming channels parallel to the [1 0 0] direction that are occupied by the bromide anions and water molecules (Fig. 2). Adjacent anions and water molecules are linked by O—H···Br hydrogen bonds, and are connected into a two-dimensional network with the cations through C—H···O and C—H···Br hydrogen interactions (Table 1).

### Experimental

1-Methylimidazole (0.14 mol) and 1-bromohexadecyl (0.15 mol) were added to a stirred solution of dichloromethane (30 ml) and stirred at 350 K for 48 h under nitrogen atmosphere. The resulting clear solution was evaporated under vacuum. Colourless crystals suitable for X-ray analysis were obtained by slow evaporation of an ethyl acetate solution over a period of two weeks (yield 83%, m.p.339.15k). Anal. Calcd (%) for C_20_H_41_Br_1_N_2_O_1_ (Mr = 405.46): C, 59.19; H, 10.11; N, 6.90. Found (%): C, 59.47; H, 9.98; N, 7.02.

### Refinement

All H atoms were placed geometrically and treated as riding on their parent atoms with O—H = 0.85 Å, C—H = 0.93–0.97 Å, and with *U*_iso_(H) = 1.2 *U*_eq_(C) or 1.5 *U*_eq_(C) for methyl H atoms.

### Crystal data

**Table:**

C_20_H_39_N_2_^+^·Br^−^·H_2_O	*Z* = 2
*M**_r_* = 405.46	*F*(000) = 436
Triclinic, *P*1	*D*_x_ = 1.170 Mg m^−^^3^
Hall symbol: -P 1	Mo *K*α radiation, λ = 0.71073 Å
*a* = 5.4989 (5) Å	Cell parameters from 2080 reflections
*b* = 7.8507 (9) Å	θ = 2.7–26.1°
*c* = 27.330 (3) Å	µ = 1.80 mm^−^^1^
α = 94.080 (1)°	*T* = 293 K
β = 91.492 (1)°	Block, colourless
γ = 101.929 (2)°	0.38 × 0.23 × 0.12 mm
*V* = 1150.4 (2) Å^3^	

### Data collection

**Table:**

Bruker SMART CCD area-detector diffractometer	3995 independent reflections
Radiation source: fine-focus sealed tube	3058 reflections with *I* > 2σ(*I*)
graphite	*R*_int_ = 0.026
φ and ω scans	θ_max_ = 25.0°, θ_min_ = 1.5°
Absorption correction: multi-scan (*SADABS*; Sheldrick, 1996)	*h* = −6→6
*T*_min_ = 0.549, *T*_max_ = 0.813	*k* = −9→9
5929 measured reflections	*l* = −26→32

### Refinement

**Table:**

Refinement on *F*^2^	Primary atom site location: structure-invariant direct methods
Least-squares matrix: full	Secondary atom site location: difference Fourier map
*R*[*F*^2^ > 2σ(*F*^2^)] = 0.057	Hydrogen site location: inferred from neighbouring sites
*wR*(*F*^2^) = 0.148	H-atom parameters constrained
*S* = 1.04	*w* = 1/[σ^2^(*F*_o_^2^) + (0.0658*P*)^2^ + 1.4252*P*] where *P* = (*F*_o_^2^ + 2*F*_c_^2^)/3
3995 reflections	(Δ/σ)_max_ < 0.001
219 parameters	Δρ_max_ = 0.58 e Å^−^^3^
0 restraints	Δρ_min_ = −0.31 e Å^−^^3^

### Fractional atomic coordinates and isotropic or equivalent isotropic displacement parameters (Å^2^)

**Table:**

	*x*	*y*	*z*	*U*_iso_*/*U*_eq_	
Br1	0.32508 (9)	0.81313 (7)	0.096948 (19)	0.0561 (2)	
N1	1.3915 (6)	0.5675 (4)	0.88874 (12)	0.0368 (8)	
N2	1.5673 (6)	0.7181 (4)	0.95356 (12)	0.0362 (8)	
O1	0.9084 (8)	0.0620 (5)	0.1247 (2)	0.1026 (16)	
H1C	0.7635	0.0007	0.1179	0.123*	
H1D	1.0139	−0.0004	0.1178	0.123*	
C1	1.5917 (8)	0.6802 (5)	0.90640 (15)	0.0396 (10)	
H1	1.7283	0.7257	0.8885	0.047*	
C2	1.3422 (8)	0.6274 (6)	0.96668 (16)	0.0435 (10)	
H2	1.2767	0.6312	0.9977	0.052*	
C3	1.2336 (8)	0.5322 (6)	0.92639 (16)	0.0425 (10)	
H3	1.0793	0.4559	0.9243	0.051*	
C4	1.3519 (8)	0.4847 (6)	0.83835 (15)	0.0444 (10)	
H4A	1.3488	0.3610	0.8393	0.053*	
H4B	1.4916	0.5341	0.8192	0.053*	
C5	1.1161 (8)	0.5065 (6)	0.81304 (15)	0.0398 (10)	
H5A	1.1131	0.6297	0.8136	0.048*	
H5B	0.9746	0.4494	0.8305	0.048*	
C6	1.0949 (8)	0.4291 (6)	0.76032 (15)	0.0432 (10)	
H6A	1.2377	0.4870	0.7433	0.052*	
H6B	1.1024	0.3067	0.7603	0.052*	
C7	0.8610 (8)	0.4429 (6)	0.73170 (15)	0.0425 (10)	
H7A	0.7178	0.3823	0.7481	0.051*	
H7B	0.8511	0.5649	0.7322	0.051*	
C8	0.8469 (8)	0.3686 (6)	0.67886 (15)	0.0431 (10)	
H8A	0.8570	0.2466	0.6785	0.052*	
H8B	0.9910	0.4289	0.6627	0.052*	
C9	0.6135 (8)	0.3815 (6)	0.64922 (15)	0.0441 (10)	
H9A	0.4691	0.3214	0.6654	0.053*	
H9B	0.6036	0.5035	0.6494	0.053*	
C10	0.6022 (8)	0.3061 (6)	0.59670 (16)	0.0439 (10)	
H10A	0.6118	0.1841	0.5966	0.053*	
H10B	0.7471	0.3659	0.5806	0.053*	
C11	0.3715 (8)	0.3186 (6)	0.56681 (15)	0.0444 (11)	
H11A	0.2265	0.2589	0.5829	0.053*	
H11B	0.3619	0.4406	0.5669	0.053*	
C12	0.3604 (8)	0.2429 (6)	0.51412 (16)	0.0453 (11)	
H12A	0.3699	0.1209	0.5141	0.054*	
H12B	0.5054	0.3026	0.4981	0.054*	
C13	0.1286 (8)	0.2556 (6)	0.48404 (16)	0.0450 (10)	
H13A	−0.0166	0.1959	0.5000	0.054*	
H13B	0.1191	0.3776	0.4840	0.054*	
C14	0.1186 (8)	0.1793 (6)	0.43125 (16)	0.0449 (11)	
H14A	0.1273	0.0572	0.4313	0.054*	
H14B	0.2642	0.2386	0.4153	0.054*	
C15	−0.1115 (8)	0.1925 (6)	0.40110 (16)	0.0449 (10)	
H15A	−0.2571	0.1335	0.4171	0.054*	
H15B	−0.1200	0.3147	0.4010	0.054*	
C16	−0.1225 (8)	0.1161 (6)	0.34835 (16)	0.0446 (10)	
H16A	−0.1132	−0.0059	0.3484	0.054*	
H16B	0.0227	0.1755	0.3323	0.054*	
C17	−0.3534 (8)	0.1286 (6)	0.31826 (16)	0.0458 (11)	
H17A	−0.4985	0.0690	0.3343	0.055*	
H17B	−0.3629	0.2506	0.3183	0.055*	
C18	−0.3646 (10)	0.0529 (7)	0.26567 (17)	0.0546 (12)	
H18A	−0.2186	0.1114	0.2497	0.066*	
H18B	−0.3577	−0.0696	0.2656	0.066*	
C19	−0.5916 (11)	0.0679 (8)	0.2361 (2)	0.0707 (16)	
H19A	−0.7376	0.0081	0.2511	0.106*	
H19B	−0.5849	0.0164	0.2034	0.106*	
H19C	−0.5978	0.1888	0.2349	0.106*	
C20	1.7530 (9)	0.8399 (6)	0.98616 (18)	0.0546 (12)	
H20A	1.9172	0.8351	0.9759	0.082*	
H20B	1.7361	0.8074	1.0193	0.082*	
H20C	1.7266	0.9565	0.9845	0.082*	

### Atomic displacement parameters (Å^2^)

**Table:**

	*U*^11^	*U*^22^	*U*^33^	*U*^12^	*U*^13^	*U*^23^
Br1	0.0480 (3)	0.0590 (3)	0.0595 (3)	0.0071 (2)	−0.0019 (2)	0.0057 (2)
N1	0.0361 (19)	0.0398 (19)	0.0343 (19)	0.0086 (15)	−0.0046 (15)	0.0022 (15)
N2	0.0325 (18)	0.0348 (18)	0.039 (2)	0.0021 (14)	−0.0084 (15)	0.0031 (15)
O1	0.059 (3)	0.064 (3)	0.178 (5)	0.002 (2)	0.025 (3)	−0.010 (3)
C1	0.031 (2)	0.046 (2)	0.041 (3)	0.0034 (19)	0.0004 (18)	0.012 (2)
C2	0.041 (3)	0.054 (3)	0.034 (2)	0.007 (2)	0.0003 (19)	0.007 (2)
C3	0.032 (2)	0.050 (3)	0.040 (3)	−0.0032 (19)	−0.0039 (19)	0.008 (2)
C4	0.044 (3)	0.053 (3)	0.038 (2)	0.016 (2)	−0.0076 (19)	−0.004 (2)
C5	0.040 (2)	0.043 (2)	0.037 (2)	0.0110 (19)	−0.0005 (18)	0.0025 (18)
C6	0.040 (2)	0.050 (3)	0.041 (3)	0.017 (2)	−0.0044 (19)	−0.005 (2)
C7	0.038 (2)	0.053 (3)	0.039 (2)	0.015 (2)	−0.0019 (19)	−0.002 (2)
C8	0.038 (2)	0.055 (3)	0.039 (2)	0.018 (2)	−0.0041 (19)	−0.004 (2)
C9	0.040 (2)	0.055 (3)	0.040 (3)	0.019 (2)	−0.0052 (19)	−0.002 (2)
C10	0.040 (2)	0.053 (3)	0.041 (3)	0.017 (2)	−0.0034 (19)	−0.003 (2)
C11	0.042 (3)	0.055 (3)	0.040 (3)	0.021 (2)	−0.002 (2)	−0.002 (2)
C12	0.041 (2)	0.055 (3)	0.043 (3)	0.017 (2)	−0.0002 (19)	−0.002 (2)
C13	0.044 (3)	0.053 (3)	0.041 (3)	0.017 (2)	−0.002 (2)	0.001 (2)
C14	0.040 (2)	0.055 (3)	0.042 (3)	0.018 (2)	−0.003 (2)	0.000 (2)
C15	0.043 (3)	0.054 (3)	0.041 (3)	0.020 (2)	−0.003 (2)	0.003 (2)
C16	0.041 (2)	0.053 (3)	0.042 (3)	0.017 (2)	−0.004 (2)	−0.001 (2)
C17	0.045 (3)	0.046 (3)	0.046 (3)	0.013 (2)	−0.005 (2)	−0.002 (2)
C18	0.058 (3)	0.063 (3)	0.044 (3)	0.018 (2)	−0.004 (2)	−0.003 (2)
C19	0.073 (4)	0.088 (4)	0.049 (3)	0.020 (3)	−0.019 (3)	−0.007 (3)
C20	0.046 (3)	0.052 (3)	0.055 (3)	−0.010 (2)	−0.015 (2)	0.000 (2)

### Geometric parameters (Å, °)

**Table:**

N1—C1	1.318 (5)	C10—H10B	0.9700
N1—C3	1.370 (5)	C11—C12	1.513 (6)
N1—C4	1.472 (5)	C11—H11A	0.9700
N2—C1	1.319 (5)	C11—H11B	0.9700
N2—C2	1.365 (5)	C12—C13	1.523 (6)
N2—C20	1.476 (5)	C12—H12A	0.9700
O1—H1C	0.8500	C12—H12B	0.9700
O1—H1D	0.8500	C13—C14	1.517 (6)
C1—H1	0.9300	C13—H13A	0.9700
C2—C3	1.339 (6)	C13—H13B	0.9700
C2—H2	0.9300	C14—C15	1.517 (6)
C3—H3	0.9300	C14—H14A	0.9700
C4—C5	1.500 (6)	C14—H14B	0.9700
C4—H4A	0.9700	C15—C16	1.517 (6)
C4—H4B	0.9700	C15—H15A	0.9700
C5—C6	1.515 (6)	C15—H15B	0.9700
C5—H5A	0.9700	C16—C17	1.519 (6)
C5—H5B	0.9700	C16—H16A	0.9700
C6—C7	1.515 (6)	C16—H16B	0.9700
C6—H6A	0.9700	C17—C18	1.511 (6)
C6—H6B	0.9700	C17—H17A	0.9700
C7—C8	1.512 (6)	C17—H17B	0.9700
C7—H7A	0.9700	C18—C19	1.499 (7)
C7—H7B	0.9700	C18—H18A	0.9700
C8—C9	1.525 (6)	C18—H18B	0.9700
C8—H8A	0.9700	C19—H19A	0.9600
C8—H8B	0.9700	C19—H19B	0.9600
C9—C10	1.508 (6)	C19—H19C	0.9600
C9—H9A	0.9700	C20—H20A	0.9600
C9—H9B	0.9700	C20—H20B	0.9600
C10—C11	1.515 (6)	C20—H20C	0.9600
C10—H10A	0.9700		
			
C1—N1—C3	108.0 (3)	C12—C11—H11A	108.5
C1—N1—C4	126.0 (4)	C10—C11—H11A	108.5
C3—N1—C4	125.9 (4)	C12—C11—H11B	108.5
C1—N2—C2	108.6 (3)	C10—C11—H11B	108.5
C1—N2—C20	125.2 (4)	H11A—C11—H11B	107.5
C2—N2—C20	126.2 (4)	C11—C12—C13	115.0 (4)
H1C—O1—H1D	108.4	C11—C12—H12A	108.5
N1—C1—N2	109.0 (4)	C13—C12—H12A	108.5
N1—C1—H1	125.5	C11—C12—H12B	108.5
N2—C1—H1	125.5	C13—C12—H12B	108.5
C3—C2—N2	106.9 (4)	H12A—C12—H12B	107.5
C3—C2—H2	126.6	C14—C13—C12	114.7 (4)
N2—C2—H2	126.6	C14—C13—H13A	108.6
C2—C3—N1	107.5 (4)	C12—C13—H13A	108.6
C2—C3—H3	126.2	C14—C13—H13B	108.6
N1—C3—H3	126.2	C12—C13—H13B	108.6
N1—C4—C5	113.8 (3)	H13A—C13—H13B	107.6
N1—C4—H4A	108.8	C13—C14—C15	114.8 (4)
C5—C4—H4A	108.8	C13—C14—H14A	108.6
N1—C4—H4B	108.8	C15—C14—H14A	108.6
C5—C4—H4B	108.8	C13—C14—H14B	108.6
H4A—C4—H4B	107.7	C15—C14—H14B	108.6
C4—C5—C6	111.2 (3)	H14A—C14—H14B	107.5
C4—C5—H5A	109.4	C16—C15—C14	115.0 (4)
C6—C5—H5A	109.4	C16—C15—H15A	108.5
C4—C5—H5B	109.4	C14—C15—H15A	108.5
C6—C5—H5B	109.4	C16—C15—H15B	108.5
H5A—C5—H5B	108.0	C14—C15—H15B	108.5
C7—C6—C5	115.3 (3)	H15A—C15—H15B	107.5
C7—C6—H6A	108.5	C15—C16—C17	115.0 (4)
C5—C6—H6A	108.5	C15—C16—H16A	108.5
C7—C6—H6B	108.5	C17—C16—H16A	108.5
C5—C6—H6B	108.5	C15—C16—H16B	108.5
H6A—C6—H6B	107.5	C17—C16—H16B	108.5
C8—C7—C6	114.3 (3)	H16A—C16—H16B	107.5
C8—C7—H7A	108.7	C18—C17—C16	115.1 (4)
C6—C7—H7A	108.7	C18—C17—H17A	108.5
C8—C7—H7B	108.7	C16—C17—H17A	108.5
C6—C7—H7B	108.7	C18—C17—H17B	108.5
H7A—C7—H7B	107.6	C16—C17—H17B	108.5
C7—C8—C9	115.2 (3)	H17A—C17—H17B	107.5
C7—C8—H8A	108.5	C19—C18—C17	114.7 (4)
C9—C8—H8A	108.5	C19—C18—H18A	108.6
C7—C8—H8B	108.5	C17—C18—H18A	108.6
C9—C8—H8B	108.5	C19—C18—H18B	108.6
H8A—C8—H8B	107.5	C17—C18—H18B	108.6
C10—C9—C8	114.5 (3)	H18A—C18—H18B	107.6
C10—C9—H9A	108.6	C18—C19—H19A	109.5
C8—C9—H9A	108.6	C18—C19—H19B	109.5
C10—C9—H9B	108.6	H19A—C19—H19B	109.5
C8—C9—H9B	108.6	C18—C19—H19C	109.5
H9A—C9—H9B	107.6	H19A—C19—H19C	109.5
C9—C10—C11	115.0 (3)	H19B—C19—H19C	109.5
C9—C10—H10A	108.5	N2—C20—H20A	109.5
C11—C10—H10A	108.5	N2—C20—H20B	109.5
C9—C10—H10B	108.5	H20A—C20—H20B	109.5
C11—C10—H10B	108.5	N2—C20—H20C	109.5
H10A—C10—H10B	107.5	H20A—C20—H20C	109.5
C12—C11—C10	114.9 (3)	H20B—C20—H20C	109.5
			
C3—N1—C1—N2	−0.2 (5)	C5—C6—C7—C8	178.6 (4)
C4—N1—C1—N2	−176.2 (4)	C6—C7—C8—C9	−179.8 (4)
C2—N2—C1—N1	−0.5 (5)	C7—C8—C9—C10	−179.8 (4)
C20—N2—C1—N1	−179.9 (4)	C8—C9—C10—C11	−179.8 (4)
C1—N2—C2—C3	0.9 (5)	C9—C10—C11—C12	−180.0 (4)
C20—N2—C2—C3	−179.7 (4)	C10—C11—C12—C13	−180.0 (4)
N2—C2—C3—N1	−1.0 (5)	C11—C12—C13—C14	−180.0 (4)
C1—N1—C3—C2	0.7 (5)	C12—C13—C14—C15	−179.7 (4)
C4—N1—C3—C2	176.7 (4)	C13—C14—C15—C16	−179.9 (4)
C1—N1—C4—C5	−126.4 (4)	C14—C15—C16—C17	179.7 (4)
C3—N1—C4—C5	58.3 (6)	C15—C16—C17—C18	179.9 (4)
N1—C4—C5—C6	176.2 (4)	C16—C17—C18—C19	−179.3 (4)
C4—C5—C6—C7	179.4 (4)		

### Hydrogen-bond geometry (Å, °)

**Table:**

*D*—H···*A*	*D*—H	H···*A*	*D*···*A*	*D*—H···*A*
O1—H1C···Br1^i^	0.85	2.58	3.429 (4)	180
O1—H1D···Br1^ii^	0.85	2.52	3.373 (4)	180
C3—H3···Br1^iii^	0.93	2.75	3.661 (4)	167
C1—H1···O1^iv^	0.93	2.38	3.231 (6)	153

Symmetry codes: (i) *x*, *y*−1, *z*; (ii) *x*+1, *y*−1, *z*; (iii) −*x*+1, −*y*+1, −*z*+1; (iv) −*x*+3, −*y*+1, −*z*+1.
